# Gene Drives in the U.K., U.S., and Australian Press (2015–2019): How a New Focus on Responsibility Is Shaping Science Communication

**DOI:** 10.1177/10755470211072245

**Published:** 2022-01-25

**Authors:** Aleksandra Stelmach, Brigitte Nerlich, Sarah Hartley

**Affiliations:** 1 University of Exeter, UK; 2 University of Nottingham, UK

**Keywords:** gene drive technology, metaphors, buzzwords, responsibility, science communication

## Abstract

Gene drive is a controversial biotechnology for pest control. Despite a commitment from gene drive researchers to responsibility and the key role of the media in debates about science and technology, little research has been conducted on media reporting of gene drive. We employ metaphor and discourse analysis to explore how responsibility is reflected in the coverage of this technology in the U.S., U.K., and Australian press. The findings reveal a rhetorical strategy of trust-building by evoking the moral attributes of gene drive researchers. We discuss the implications of these findings for the communication of new technologies.

## Introduction

Gene drive is an emerging biotechnology designed to control pests, including invasive rodents and insect pests in global health, conservation, and agriculture. It differs from traditional genetic modification in that a modification is designed to be driven through a population, potentially changing the whole species. Although gene drive technology is at an early stage of development, it has attracted controversy and led to calls to ban its development as it is considered high risk (Callaway, 2018). Researchers involved in this new field have been keen to highlight the importance of transparency and openness when doing their research and have publicly stated their commitment to responsibility (Emerson et al., 2017; Esvelt, 2016; Long et al., 2020). This public demonstration of their commitment to responsibility has involved the media in communicating what gene drive technology is, what it can do, what risks it involves, and how their research is carried out responsibly. In addition, gene drive researchers are acutely aware of the power of language in shaping public support for their technology and have taken active steps to shape this language (Alphey et al., 2020).

The media are a key site where science and technology are debated and legitimized—or de-legitimized—through the use of language with important implications for influencing social attitudes and understanding (Kitzinger & Williams, 2005). Public debates about novel and contested technologies usually revolve around the issues of risk and ethics (Kastenhofer, 2009), two prominent lenses alongside that of benefits through which technologies are given a particular meaning (Bogner & Torgersen, 2015). In the context of nanotechnology and synthetic biology, debates on ethics have tended to focus on the issues of responsibility in the conduct of scientific research and innovation, and they were often entangled with issues of risk as well as benefits (Bauer & Bogner, 2020; Kelty, 2008). The two concepts of risk and responsibility are so closely linked that, as McCarthy and Kelty (2010) argue, they should be considered as a pair: “wherever risk is salient, responsibility is its implicit shadow” (p. 407). How the notions of responsibility and risk as well as benefits are discussed in the public realm, and which of them are emphasized or de-emphasized, can shape the perception of a technology as a potential source of conflict over its development and future use (Torgersen & Schmidt, 2013).

Although contributions to science debates in the media have become more pluralistic and now include a wider range of voices (Schäfer, 2009), scientists maintain considerable influence over what is being said about science and how (Bubela & Caulfield, 2004; Nelkin, 1987). This is because scientists remain a primary source of scientific information in the press (Peters, 2013), and they use the media to advance their political agenda, especially during controversies (Brossard, 2009). This influence is compounded by pressures on scientists to strategically use the media to disseminate their findings and demonstrate the social relevance and responsible conduct of their research (Nerlich & McLeod, 2016; Weingart, 1998).

However, the concept of responsibility and responsible research, despite being widely debated in academic literature, remains vague. While it is now considered as part of international discussion in academia and policy circles, it remains open to interpretations, especially as to how it could be put to practice by scientists themselves (Davies & Horst, 2015; Glerup & Horst, 2014). Responsibility, as Kelty (2008) put it, is “not quite ethics, not quite safety, not quite duty or vocation, but somehow related to them all. What concept might be associated with this term is never very clear” (p. 1). Studies of the ways in which scientists interpret responsibility and of how they try to make it “doable” have started to shed light on how it is being understood and put in practice in the context of specific technologies (Corley et al., 2016; Loroño-Leturiondo & Davies, 2018; McCarthy & Kelty, 2010). It remains unclear, however, how it is being communicated in the media and to lay audiences. The emerging research in this area indicates that public debates of responsibility are characterized by a high level of abstraction and are focused on generic issues, such as the value of ethical reflection for the development and governance of new technologies (Bauer & Bogner, 2020).

Gene drive^1^ is an area of research where the issues of risks, benefits, and scientific responsibility have stimulated much debate, not least as it promises to help control vector-borne diseases and to manage invasive species (Burt, 2003). Gene drives are “engineered snippets of DNA that can be introduced into an organism’s genome to significantly increase the chance that a desired genetic trait will spread through a population faster than would normally happen through sexual reproduction,” even though it is not beneficial for the species (Friedman et al., 2020, p. 72). Current research efforts focus on malaria suppression or eradication (Scudellari, 2019) and the eradication of nonnative species such as feral cats, foxes, and rodents that threaten the survival of native birds and mammals (Edwards et al., 2017). In the prevention of malaria, genetic engineering could make mosquitoes less likely to transmit the plasmodium parasite which causes malaria in humans (Buchman et al., 2020; Scudellari, 2019). Alternatively, gene drives could disrupt reproductive functions of targeted organisms, rendering female mosquitoes unable to bite and lay eggs (Kyrou et al., 2018), or causing insects such as mosquitoes, or mammals such as feral cats, to produce mainly male offspring, thus reducing the population of the targeted species (Edwards et al., 2017; Kachel, 2018).

While research on gene drive is largely confined to laboratories and gene drive organisms have not been trialed in the wild, the technology remains controversial. Uncertainty exists about whether it will work, how effective it might be, who is controlling it, and potential unintended consequences for human health and the environment (European Network of Scientists for Social and Environmental Responsibility, 2019; Webber et al., 2015). Aware of these uncertainties and concerns, the gene drive community, including scientists, funders, and supporters of the research are committed to conducting research in a transparent fashion (Esvelt et al., 2014; James & Tountas, 2018; Ledingham & Hartley, 2020; National Academies of Sciences, Engineering, and Medicine [NASEM], 2016; Oye et al., 2014). For example, a statement from a group of gene drive researchers declared that “we must ensure that trials are scientifically, politically, and socially robust, publicly accountable, and widely transparent. [. . .] We pledge [. . .] to contribute to a fair and ethical culture of gene drive research” (Long et al., 2020, pp. 1417–1418). Prompted by this proactive community, core research and ethical discussions started to be reported in popular media where key figures from gene drive research, such as Kevin Esvelt from MIT, talked about scientific responsibility (see, for example, HBO, 2018; Netflix, 2019).

Given the potentially transformative nature of gene drive as well as its capacity to elicit controversy, fear, and mistrust (Singh, 2019), the ways in which it is discussed in the media can shape early public understandings, attitudes, and ultimately support for this technology. Examining linguistic features of the coverage can uncover polarizing views and shed light on the dynamics of the public debate and communication strategies adopted by various stakeholders (Petersen, 2005), including gene drive developers as well as opponents of this technology. Very little research has been conducted on how the issue of scientific responsibility as well as risks and benefits of gene drive have been reported in the media. A study by Kamenova and colleagues (2017) has shown, for example, that gene drive has been portrayed as a high-risk technology affecting human populations and the environment with little or no mention of responsibility. Identifying this gap, Brossard et al. (2019) highlight the need for more research on how gene drive technology is communicated in the media. Overall, little is known, for example, how language is used in the media coverage, and how discursive devices, such as metaphors, are deployed to shape understandings of this technology. Metaphors and stories, or “biofantasies” (Petersen, 2001), are especially important at the early stage of technology development, when the exact science of gene drive is still taking shape and actors are trying to make sense of the science and its meaning. Metaphors also provide a lens through which the issues of scientific responsibility (Döring, 2018) as well as risks and benefits (Petersen, 2005) of emerging technologies are negotiated in the public realm. It is likely that metaphors, as well as other discursive devices such as clichés, hyperbole (Nerlich et al., 1999), and buzzwords (Bensaude Vincent, 2014) are used to communicate certain aspects of the science, what it can achieve, and the possible risks or ethical concerns.

We address this gap through a discourse analysis of media coverage of gene drive research, including an analysis of discursive devices, such as metaphors and buzzwords. We analyze media reporting between 2015 and 2019 in the three countries that are world leaders in gene drive research: the United Kingdom, the United States, and Australia. The work on gene drive mosquitoes for malaria control is most advanced in the United Kingdom and the United States, while scientists in Australia are leading in research on gene drive mammals for conservation (Scudellari, 2019). We examine the discursive devices used in gene drive communication and explore the way in which responsibility, risks, and benefits are reflected in linguistic features of newspaper articles. In this study, we aim to examine not only what meanings are carried by metaphors and other discursive devices but also how they interact and how they function rhetorically in the media coverage. Our research has important implications for the communication of emerging science and technologies, where the importance and commitment to responsibility is increasingly evoked (Bauer & Bogner, 2020), but its performative significance has not been yet explored.

## Theory and Method

This study is located within the field of discourse and metaphor analysis which studies how language is used in scientific communication, and what effect it has on debates and understandings of scientific fields (Maasen & Weingart, 1995; Myers, 2003). Much of this research has focused on controversies surrounding emerging biotechnologies, where metaphors shaped the debates about risks, benefits, and ethical issues surrounding genetically modified (GM) food, synthetic biology, and gene editing, to name just a few (Cook, 2004; Hellsten & Nerlich, 2011; Nelson et al., 2015).

Metaphors are pervasive in every aspect of science, from theory building to science communication. They allow the capture of new, unfamiliar, and abstract phenomena in terms of more concrete subject matters, and they foster understanding of complex issues by referring to concepts and objects from everyday experience (Larson et al., 2005). Metaphors are also powerful rhetorical devices that can be used to support three classical strategies of persuasion: the appeal to logical arguments (*logos*), the appeal to emotions (*patho*s), and appeals to the good character of the speaker and the shared values (*ethos*) (Ferrari, 2018; Miyawaki, 2018). Appeals to emotions as well as to good character and trustworthiness are especially relevant for the studies of controversies, where opposing sides use a range of discursive tools to legitimize or de-legitimize a particular cause (Simon, 2020).

Of particular interest are metaphors of war and destruction, which are often used to convey hopes and fears, or risks and benefits of emerging fields. They are ubiquitous in some areas of science and medicine, especially in conservation biology, pest management, and control of infectious diseases (Larson et al., 2005). These are also the fields in which applications of gene drive technologies are being considered. Studies have shown that metaphors of war have important rhetorical role: They are effective in attracting attention, but their use is controversial as they evoke fear, draw on hyperbole, and amplify perception of urgency and threat among lay publics (Flusberg et al., 2018). They can polarize opinion and lead to conflicts over solutions to the problem among various activist groups, while the excessive focus on control evoked by war metaphors can also encourage radical solutions (Larson, 2005).

Going beyond the customary analysis of metaphors, this study also examines the role played in scientific communications by other discursive devices, such as hyperbole, simile, clichés, and especially buzzwords. Unlike metaphors which received extensive attention in research on the language of science debates, the use of buzzwords remains understudied (Bensaude Vincent, 2014). Buzzwords, words considered fashionable for a period of time, and clichés, words that used to be fashionable but became overused, are a staple of both specialist writings and scientific popularizations (Capoor, 2017; Cornwall, 2007; Nerlich et al., 1999). Rather than being mere stylistic devices, they have an important performative function. They help claim authority, facilitate action, and displace responsibility for hard decision, and they can signal ethical commitments and be used as “moral badges,” as research in management and development has shown (Cluley, 2013; Dahl, 2008). In this study, we draw in particular on Bensaude Vincent’s (2014) insights into the performative function of buzzwords such as “public engagement in science” and “responsible innovation.” As linguistic units with poorly defined or “fuzzy” meanings, buzzwords act like slogans and have a mobilizing role: They highlight matters of concern, build consensus around these matters, and bring people together by outlining inspiring goals and agendas. Rhetorical uses of metaphors and other discursive tools deserve a critical analysis, as they can shape beliefs and attitudes toward science, and encourage or discourage a particular course of action (Bono, 2001).

### Sample and Analytical Procedure

We analyzed media coverage in the United Kingdom, the United States, and Australia, countries which are at the forefront of gene drive research. We concentrate on major news sources, including elite media, which play an agenda-setting role in defining what is newsworthy (Nisbet & Lewenstein, 2002) and which remain a trusted source of information about science among lay publics (Department for Business, Energy and Industrial Strategy, 2019).

**Figure 1.**
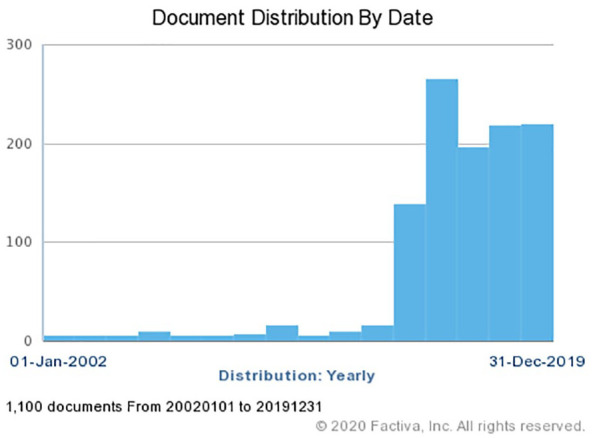
The rise of reporting on “gene drive” in English-language media. *Source*. Factiva, search period between January 1, 2002, and December 31, 2019.

We used the media database Factiva to search for articles on “gene drive” published in the U.S., U.K., and Australian media between January 1, 2015, and December 31, 2019. This period captures the rise of scientific and media attention to gene drive between 2015 and 2019 (Figure 1). The “source category” was “Major News and Business Sources,” which includes media outlets with the highest circulation and also enjoying considerable prestige, such as *The New York Times* and *The Washington Post* in the United States, *The Times* and *The Independent* in the United Kingdom, as well as *The Australian* and *The Canberra Times* in Australia. We used a Factiva function to browse the database with the exact search phrase “gene drive” to avoid confusion with other terms, such as “cancer gene drivers” (see also Nerlich, 2019) and to reduce the number of irrelevant pieces. After removing duplicates, 159 articles remained in the sample, including 67 articles for the United States, 78 for the United Kingdom, and 14 for Australia. The Australian sample is much smaller than the other two. These sample sizes are not surprising given that gene drive is an emergent technology.

Our analysis drew on Lakoff and Johnson’s (1980) theory of metaphor, which distinguishes between conceptual metaphors (usually represented in small capitals) and the linguistic expressions that can be derived from them. For example, Life is a journey is a conceptual metaphor which maps aspects of journeys onto how we conceptualize life, while “we have reached the end of the road” or “my life is at a cross roads” are verbal metaphors based on this conceptual metaphor. This approach to metaphor analysis has been successfully applied to the study of scientific texts and popularizations. As Nelson et al. (2015) have made clear,

Metaphor analysis involves identifying metaphorical language and then articulating the underlying metaphorical concepts [. . .]. For example, phrases such as “the genome is read” and the “first draft of the human genome” can be grouped into the underlying metaphorical concept “the genome is text.” (pp. 60–61)

We examined the conceptual metaphors underlying discourses about gene drive and their expressions at the level of media representations of this technology.

We combined metaphor and discourse analysis with thematic analysis, which relies on identifying, analyzing, and reporting patterns (themes) within data (Braun & Clarke, 2006, p. 78). Salient themes often cluster around salient metaphors and other discursive features (Bernard et al., 2009; Cassell & Bishop, 2018). We focused on single newspaper articles as the unit of analysis. The first two authors read and re-read the articles to familiarize themselves with the broader themes that we subsequently discussed analytically as a team. First, we made initial observations on the essential qualities of each article and dominant rhetorical techniques. Second, we discussed our respective initial codes, which included general tone, forms of language, comparisons, categorizations, and emerging patterns in the data. Third, the initial codes were collated into preliminary themes and subsequently arranged into a coherent structure. The two coders met regularly during the coding process to compare their findings, and any uncertainties or disagreements were subsequently discussed and assessed in team meetings to achieve consensus. In addition to describing dominant themes in the corpus, we identified linguistic elements, especially metaphors, which performed the functions of familiarizing readers with gene drive or tried to shape readers’ expectations, hopes and fears regarding this emerging technology. We also focused on other discursive features, especially buzzwords, and examined how they conveyed the issues of responsibility in relation to gene drive research. We paid attention to patterns in the media coverage, including typical examples as well as exceptions and contradictions, which allowed for a deeper exploration of media discourses and provided broader insights into representations of gene drive (Macnamara, 2005; Miles & Huberman, 1994).

## Results

Across the media sample, the coverage stayed close to scientific and institutional sources, and it relied heavily on scientists at the forefront of gene drive research. The most visible of these were Kevin Esvelt (MIT), as well as Austin Burt and Andrea Crisanti (both at Imperial College London). In contrast, there were no “visible scientists” (Goodell, 1977) in our small Australian sample. The Bill and Melinda Gates Foundation also featured in press reporting, heavily in the United States, and to a lesser extent in the United Kingdom and Australia. This philanthropist organization funds Target Malaria, a research consortium based at Imperial College London and developing gene drive mosquitoes for malaria control in sub-Saharan Africa. Target Malaria is expected to be the first actor to trial a gene drive organism (Hartley et al., 2019). Other actors quoted in the press were health and conservation experts, international organizations such as the World Health Organization and the United Nations Convention on Biological Diversity, industry, bioethicists, and leaders of nongovernmental organizations (NGOs), some of which, such as the ETC Group, GeneWatch, and Friends of the Earth, oppose gene drive.

Our analysis revealed four main themes in the media coverage: (a) the nature and mechanisms of gene drive; (b) the hopes related to potential benefits of using gene drive; (c) fears, risks, and unintended consequences of using gene drive in the wild; and (d) the role of scientists driving gene drive. These themes were discussed in the media with the help of discursive devices, most importantly metaphors, but also clichés, simile, hyperbole, and buzzwords. The media in the United States and the United Kingdom, where coverage about gene drive was more extensive, used the most metaphors and the most diverse metaphors and discursive tools. These themes are discussed in turn below.

### The Nature and Mechanisms of Gene Drive: Explaining Through Clichés and Metaphors

Newspaper articles drew on a range of discursive tools to explain the science and the mechanisms of gene drive. The cliché of “revolutions” and “breakthroughs” was used to convey the promissory nature of this emerging field. For example, *The New York Times* described gene drive as a “revolutionary genetic technique” that offered control over insect-borne diseases such as malaria (December 22, 2015). In Australia, gene drive was hailed as “a real breakthrough with feral cats” that threaten the survival of many native species of birds and mammals (*The Australian*, June 11, 2018).

The control over nature provided by gene drive technology and the possibility to alter entire species was conveyed through the conceptual metaphor Evolution is design. It linked the theory of evolution with the new technology which now enabled scientists to modify the make-up of entire populations of animals and to change the course of their evolution. This was explained through metaphorical expressions which explored the power of scientists to “assist” the evolution or to “bend it to our will” (*The New York Times*, November 10, 2015), to “sculpt” (*The Boston Globe*, August 22, 2019) or even “reverse” it (*The Telegraph*, August 11, 2018). At the same time, the media emphasized the harmful properties of genetic traits designed to spread rapidly within populations of disease-carrying mosquitoes or invasive species. This was conveyed through metaphorical expressions and simile which depicted gene drive as engines or diseases spreading harmful modifications. For example, it was described as a “technique to spread ‘supercharged’ genes” (*The Advertiser*, August 5, 2015), or a technology “which enable[s] GM genes to spread rapidly like a viral infection within a population” (*The Independent*, August 3, 2015).

Despite the promise of power over nature, the coverage was not unanimously optimistic. Instead, the media displayed an ambivalence about gene drive, highlighting its potential advantages and pitfalls. This ambivalence was reflected in wordplay, such as “dangerous driving” (*The Age*, February 24, 2018), as a “powerful double-edged technology” (*The Pittsburgh Post-Gazette*, December 20, 2015), or as “a power for good or evil” (*The Independent*, August 2, 2015). Overall, the metaphors and clichés explaining what gene drive is and how it works drew on the well-trodden tropes of control over nature and evolution, but did not convey an unwarranted optimism. The language of breakthroughs and promises overlapped with the one of risks and uncertainties. The focus on risk or danger was also evident in the representations of hopes and fears conveyed through metaphorical clusters of destruction and apocalypse, as we show next.

### The Hopes and Benefits of Gene Drive: Endorsement Through War Metaphors

The hopes for potential applications of gene drive were expressed in aggressive metaphors denoting war, conflict, and destruction. This type of language was pervasive in the reporting across all the countries we studied. This is not surprising as war metaphors are almost automatically triggered when talking about pest control and disease management (Larson et al., 2005). War metaphors highlighted both the efficiency of the gene drive technology and the seriousness of the problems to which it offered a solution. They were used to depict the damage inflicted on humans and the environment by disease-carrying insects and invasive species, and also to convey the damage gene drive could, in turn, inflict on the insects and pests. As such, war metaphors acted as tools of persuasion to advocate deploying gene drive in public health and conservation. War metaphors were used by different stakeholders: journalists, experts, and NGOs drew on the imagery of destruction to get their point across in a forceful way.

The use of war metaphors is linked to well-established conceptual metaphors exploited in discourses of disease and invasive species management, such as Managing disease is war (with other metaphors spinning out from there, such as insects/pests are enemies; Larson et al., 2005). The metaphorical expressions of war identified in the coverage ranged from routine to hyperbolic. The routine ways of talking about tackling pest or disease involved mentions of elimination, eradication, killing, fighting, battling, wiping out or annihilating the targeted species. In the media coverage, we found examples of such unreflective talk, such as “fighting invasive species” (*The Independent*, August 02, 2015), and “wiping out a species” (*Newsweek*, December 11, 2015), as well as more hyperbolic ones, such as “epic annihilation” (*The Times*, May 18, 2018) and “perennial battle between humans and mosquitoes” (*The New York Times*, January 31, 2016). Some articles drew explicitly on declarations of war, such as “Mosquitoes, this time it’s war” (*USA Today*, February 4, 2016), “war against invasive zebra mussels” (*The Star Tribune*, July 31, 2017), or “gene war strategy to rub out feral cats” (*The Australian*, May 25, 2018).

The urgency of using this technology was emphasized through expressions which personified animals as villains (Lynteris, 2019). Thus, the media, as well as various actors quoted in the media, talked about “killer mice” (*The Financial Times*, May 27, 2016), “supercharged killer mosquitoes” (*The Daily Star*, August 5, 2015), or a mosquito as “man’s deadliest enemy” (*The Telegraph*, August 11, 2018). An extreme example of such coverage and hyperbole appeared in Australian media where feral cats and foxes were accused of “genocide” of native species, and of turning Australia into “a marsupial graveyard” (*The Weekend Australian Magazine*, May 26, 2018).

There were exceptions to such representations. Instead of highlighting the harms inflicted by wild insects or pests, some media reports emphasized the benefits of genetically engineered animals. For example, the gene drive technique that makes mosquitoes less likely to transmit malaria prompted media reports on “malaria-proof mosquitoes” (*St. Louis Post-Dispatch*, June 12, 2016), “anti-disease mosquitoes” (*The Australian*, November 23, 2015), or “risk-free mosquitoes” (*The Telegraph*, August 11, 2018). This linked gene drive mosquitoes to antimalarial drugs or bed nets, and described them as public health tools for controlling infectious diseases (Beisel & Boëte, 2013).

Overall, the press drew on an aggressive language to convey hopes related to gene drive. War metaphors and hyperbole derived from well-entrenched discourses of war on disease and invasive species highlighted the urgency of problems in public health and conservation, and the effectiveness of this technology. Even when used routinely in the media, war metaphors had a persuasive function, as they rhetorically mobilized support for this technology and called for elimination of diseases and invasive species. However, aggressive language and metaphors were also used in the media to warn against using gene drive, as we show next.

### Fears, Risks, and Unintended Consequences: Contesting Gene Drive Through Scare Metaphors

Fears about gene drive technology clustered around unforeseen consequences of using it in the wild, and they focused on the damage that it could inflict on both humans and the environment. These fears were conveyed by what the zoologist and historian of medicine, Matthew Cobb, recently called “scare metaphors” (Nerlich, 2020)—hyperbolic metaphorical expressions denoting extreme power and danger.

Scare metaphors evoking the threat of nuclear weapons and ecological apocalypse stood out in the media coverage across our sample. They were derived from the specialist term “mutagenic chain reaction,” first used by scientists Gantz and Bier (2015) to metaphorically describe the mechanism of gene drive and to emphasize the speed and efficiency with which engineered genetic elements spread through the population of animals (NASEM, 2016). Such ways of talking are common in science—as in polymerase chain reaction (PCR), for example. But when used in popular media, the jargon term “mutagenic chain reaction” took on more potent metaphorical meanings. While it was initially used to simply describe the gene drive technology, it soon came to define its dangers and was picked up by its opponents.

In the press, a wide range of metaphorical expressions were underpinned by the conceptual metaphor gene drive is a nuclear weapon. Opponents of gene drive were particularly active in promoting the idea that gene drive technology constitutes a kind of biological bomb. The most prominent example involved the campaign conducted by the ETC Group. In 2016, it issued a call to “Stop the Gene Bomb!” (*The Washington Post*, June 9, 2016), which used the metaphor of mutagenic chain reaction to arouse fear and advocate for a moratorium on this technology. As Jim Thomas, program director of the ETC group, argued, “a gene drive might be better regarded as a ‘*gene bomb’: dropped* into the normal course of inheritance, it *annihilates* natural variety (. . .). It may even *annihilat*e the species itself” (*The Guardian*, June 10, 2016, italics ours).

The notion that gene drive is a bomb was a recurring feature of the coverage, especially at the beginning of our reporting period. Gene drive technology was described as “a sort of genetic time bomb that could wipe out the species” (*The Wall Street Journal*, July 7, 2017), or as “genes that act as a time-delayed bomb in the population” (*The Times*, July 25, 2018). Although the metaphor of the nuclear bomb eventually died out in the coverage, the image of gene drive as a destructive technology was supported by a web of other metaphorical expressions, such as “genetic extinction technologies” (*The Guardian*, December 4, 2017), a “tool that spreads catastrophic mutations” (*The Times*, September 25, 2018), or “exterminator technology” (*The Washington Post*, December 2, 2018).

The destructive power of gene drives was also highlighted by the metaphorical expressions evoking terrorism. The U.S. media speculated about the use of gene drive as a “terrorist bioweapon” (*The Christian Science Monitor*, December 18, 2015), especially after James Clapper, former Director of National Intelligence, had listed it as “a potential weapon of mass destruction” (*The New Yorker*, January 2, 2017). In the United Kingdom, the focus was on the threat of bioterrorism and the fear that “mozzies” could become a “new ISIS weapon” (*The Daily Star*, August 5, 2015), and to a lesser extent on gene drive use by the military, the prospect raised, for example, by the ETC Group (*The Guardian*, June 10, 2016).

Other risks, such as unforeseen consequences for the animals or ecosystems, also prompted speculations about disasters, especially in the U.K. and U.S. coverage. For example, describing the impact of mosquito eradication on food chains, *The Guardian* claimed that a “total mosquito apocalypse would be a catastrophe” (*The Guardian*, February 10, 2016). A piece in *The Washington Post* warned that “Without proper caution . . . gene drives could deliver ecological disaster . . . we could unleash monsters” (November 25, 2015). Things were different in Australia where catastrophic language and metaphors dominated the coverage of the impact of invasive animals on native species, thus reinforcing the case for the use of gene drive in conservation.

Overall, scare metaphors and imagery of a nuclear bomb, bioterror, secret military deployment, and ecological disasters denoted the fears and mistrust of the gene drive technology, and were used as a rallying cry against its use. While some of these metaphors originated in scientific and security circles and were initially intended as more specialist terms, they were picked up by the media, where journalists, as well as various experts or opponents of gene drive used them to warn against this technology.

### The Role of Scientists Driving Gene Drive: Legitimizing Gene Drive Through Buzzwords

Although the press drew on striking linguistic devices to emphasize the transformative potential and dangers of gene drive, their depictions of the scientists behind this technology differed remarkably in the use of language. Few, if any, metaphors were used to describe the scientific community. Instead, the coverage emphasized the way in which scientists were said to conduct their research, highlighting key attributes such as responsibility and caution, transparency and openness, as well as commitment to public engagement and shared public values. While the meanings of these attributes were not clearly defined, they were evoked or repeated in various contexts describing scientific conduct or the interface of science and society. As such, the notions of responsibility, caution, and other characteristics acted like buzzwords which often drew their meanings from context (Bensaude Vincent, 2014).

The press described the gene drive community as transparent about their research. The insistence on transparency and openness characterized scientists’ and media descriptions about scientists from the very start. For example, across the three countries we studied, the media reported on a prominent letter published in *Science*, in which scientists, including gene drive developers, advocated public debate and called “on the scientific community to be open and transparent about both the risks and benefits of gene drives” (*The Canberra Times*, August 8, 2015). This created an image of gene drive researchers self-imposing new ethical norms of scientific conduct on their own community.

The emphasis on scientific transparency was most salient in the United States, where Kevin Esvelt became associated with a new way of doing and communicating science. In a popular essay, Esvelt (2018) argued that openness was the only way to dispel fears about this new technology. These and similar assertions of scientific transparency filtered from specialist into popular media, where Esvelt was quoted as saying, for example, that “for both moral and practical reasons, gene drive is most likely to succeed if all the research is done openly” (*The New Yorker*, February 2, 2017). The need for transparency was thus emphasized not only as a moral duty but also as a pragmatic strategy for winning public support.

There were a few exceptions to the treatment of transparency in the press. In Australia, reports about Defense Advanced Research Projects Agency (DARPA) funding for gene drive sparked fears that controversial research on genetically engineered rodents was being conducted without public scrutiny (*The Age*, February 24, 2018). In the United Kingdom and the United States, NGOs opposing gene drive, such as GeneWatch and the ETC Group suggested that developing gene drive for malaria control could in turn lead to its widespread use in commercial applications, and without public oversight (*The Independent*, August 3, 2015).

On the whole, however, media coverage supported the image of scientists as trustworthy, transparent, and not hiding inconvenient facts. In the press, scientists were the key source of both hopeful statements and warnings about risks, a finding also reported by Kamenova et al. (2017). In the coverage, the words “warn,” “alarm,” or “fear” were used to describe experts’ pronouncements on risk and unintended consequences of gene drive. Not only did the scientists warn about the potential dangers but they also advocated responsibility and caution in the conduct of research, thus aligning themselves with the precautionary approach favored by main scientific and regulatory bodies such as NASEM. Cautioning about potential unforeseen consequences and weighing risks and benefits was described as a scientific duty and as a “responsibility that surely no biologist takes lightly” (*The New York Times*, December 22, 2015).

Newspaper articles not only presented scientists as advocating responsibility and caution but also as already doing research in a responsible way. In contrast to previous debates about biotechnologies which promised imminent cures and solutions (Kitzinger & Williams, 2005), gene drive developers quoted in the press were keen to emphasize the slow and cautious progress of research. For example, the work of Anthony James of the University of California was described as proceeding “in careful stages with the knowledge and approval of local authorities” (*The New York Times*, November 25, 2015). Similar accounts of scientific caution appeared in the U.K. press where Andrea Crisanti was quoted as saying, “There is a risk we all rush, and that shouldn’t be done. [. . .] And we shouldn’t take shortcuts in the regulatory process. We don’t want it to backfire when it could be a gamechanger for public health” (*The Guardian*, February 10, 2016).

Scientists were represented not only as conducting research and providing expert opinion but also as engaged in activities demonstrating their social responsibility (Douglas, 2003). This involved signing open letters, issuing calls for public debates, supporting research safeguards, and advocating for a greater engagement of lay publics in the process of decision-making about the future use of gene drive. Most prominent accounts of such activities appeared again in the United States where Esvelt’s attempts to engage local communities on the island of Nantucket in his research were followed in great detail by *The New York Times* and the *Boston Globe.* In the United Kingdom as well, scientists speaking to the media advocated the need for public engagement and consent for the potential use of gene drive. For example, Austin Burt, who leads Target Malaria, argued in the press that his research consortium “is advancing cautiously, consulting widely and being as transparent as possible. Its job is simply to develop the tool and let others decide whether to use it, he says . . . It’s up to the Africans to decide” (*The Telegraph*, August 11, 2018).

The idea that decisions about gene drive were ultimately in publics’ and policy makers’ hands recurred in the coverage, but—apart from the accounts of Esvelt’s public engagement activities—it tended not to be discussed in much detail. Instead, it was often subsumed in general statements, such as, “There are many unknowns and potential risks must [be] discussed transparently in public forums” (*The Sydney Morning Herald*, April 22, 2019).

Only rarely was doubt cast on scientific responsibility and competence. Occasionally, articles made reference to scientific irresponsibility and hubris, conveyed by the metaphor of “playing god” or by stock book titles such as “Frankenstein,” “Jurassic Park,” and “sorcerer’s apprentice.” These expressions were deployed in past debates about GM food or cloning to raise doubts about these technologies and scientists’ motivations (Cook, 2004; Nerlich et al., 1999). In gene drive reporting, such rhetorical devices were used, for example, by Dana Perls from the gene drive-opposing group Friends of the Earth who urged that “We should not be playing God in the garden with things scientists admit they do not fully understand” (*The Telegraph*, August 11, 2018). The stock book title of the “sorcerer’s apprentice” was used in an article in which Ali Tapsoba from the nonprofit organization Terre à Vie opposing gene drive trials in Africa accused Target Malaria of “medical colonialism”: “If Bill Gates [who funds Target Malaria] wants to help us, then he should ask us what we want, not do something we don’t want” (*The Guardian*, December 3, 2018). These were rare instances in which the talk about scientific responsibility and democratic commitments was challenged by other stakeholders. Future research could usefully investigate challenges to the notion of scientific responsibility from diverse communities, especially when gene drive research enters the stage of field trials.

Overall, scientists were represented as trustworthy, transparent, and taking their social responsibilities seriously. Rather than talking about the need to educate lay publics, scientists quoted in the articles emphasized the need to work with publics, and stressed their commitment to listen and address people’s concerns (Hartley et al., 2019; Ledingham & Hartley, 2020). With a few exceptions, they also avoided grand statements and adversarial language, adopting the language of humility rather than of hubris (Jasanoff, 2005).

## Discussion

This study set out to analyze metaphors and discursive tools used in the media to discuss the science and controversies surrounding gene drive technology. It sought to examine if and how the notions of scientific responsibility and caution are reflected in the linguistic features of media reporting. Our study uncovered competing strategies for discussing this technology. In particular, it identified a strategy of eliciting trust in the gene drive technology by invoking moral attributes of the gene drive community, notably scientific responsibility and caution. We reveal four major themes that dominated reporting of gene drive: the science of gene drive, hopes as well as fears related to this technology, and the role of the gene drive community. Each of these themes was conveyed through a range of metaphors, clichés, buzzwords, and other discursive tools. These linguistic devices played a rhetorical function and appealed to logical arguments, emotions, or the good character of scientists and shared values.

Persuasive arguments about the science of gene drive were expressed with the help of clichés of breakthroughs and metaphors of control over evolution. These discursive tools are well established in standard accounts of scientific fields which draw on the tropes of progress and mastery over nature and which emphasize the expertise and credibility of scientists (Nerlich et al., 2003). Journalists and experts resorted to this way of talking about gene drive to highlight the novelty and the excitement caused by this research, as well as the ambivalence toward its potential consequences.

Appeals to emotions were pervasive in the media coverage. Both proponents and opponents of gene drive used emotive language to get their point across in a forceful way. On the one hand, hopes that gene drive could provide a solution to pressing problems such as malaria and invasive species were expressed in an aggressive language, notably in metaphors of war which are well-established ways of talking about pest and disease control. War metaphors emphasized the urgency of global health and conservation issues, highlighted the efficiency of gene drive, and sought to mobilize support for this technology. On the other hand, discursive devices evoking fears and mistrust of gene drive were also used by opponents of gene drive to spur resistance and to delegitimize the research. This happened through hyperbole, simile, and scare metaphors which conveyed the threat of nuclear disaster, bioterrorism, secret military deployment, and ecological apocalypse. Such catastrophic language is also a well-known strategy to attract attention to potential risks (Weingart, 1998).

Finally, this analysis reveals a strategy of promoting the moral authority of the gene drive community. Rather than focusing solely on scientific competence which confers credibility and respect to scientists as experts in their field, the media accounts highlighted other trust-building attributes (Fiske & Dupree, 2014), which related to the social responsibility of scientists and their commitment to shared public values (Douglas, 2003; Glerup & Horst, 2014). This was discursively achieved largely without metaphors and through repeated emphasis on responsibility, caution, transparency, the willingness to be truthful, and commitment to democracy and public engagement. While each of these notions was rather fuzzy and poorly defined in the media coverage, together they formed clusters of meaning that created “buzz” (Bensaude Vincent, 2014) and performed the role of building trust in scientists and consensus around the gene drive technology. In addition, the media presented scientists as demonstrating their social responsibility not just through words but also through actions. This was done through accounts of scientists signing open letters, calling for caution, and advocating public engagement. Journalists, as well as scientists speaking to the journalists about their own work were engaged in the promotion of these key moral attributes of the gene drive community.

Our article demonstrates the importance of studying communication at an early stage of technology development, when various actors attempt to influence the debate and public understanding of gene drive despite uncertainties surrounding this technology. The gene drive community has been active in shaping the language of gene drive to engage lay publics and to build trust (Alphey et al., 2020; Cheung et al., 2020; Schairer et al., 2020), and our study is one of the first to examine how language is used to convey the issues of risks, benefits, and scientific responsibility in media debates on gene drive. It shows how such strategies of building support and trust for gene drive are deployed in the public arena and mediated through elite press reporting. It is important to stress that this analysis explores the performative functions of the notions of responsibility and caution used as buzzwords in the media coverage. It does not evaluate the scientific community’s responsibility and/or caution.

Our findings indicate an ongoing struggle over the language of the gene drive debate. The dominance of aggressive metaphors of war remains entrenched, particularly in the promotion of solutions to global health and conservation challenges which also reflect opponents’ motivations to stop this technology by arousing fears of its potentially catastrophic consequences. Yet, these familiar ways of reporting are juxtaposed against a new focus on responsibility where the language of controversy and war is muted by the language of consensus, specifically by the use of buzzwords evoking the social responsibility of scientists and their commitment to shared values. As Bensaude Vincent (2014) has noted, successful buzzwords “act as pacifiers: they aim to overcome dissent” and “they convey the view of a consensual world that is built up through a soft and diplomatic process of negotiation” (p. 246). They also have the potential to “depoliticize” emerging technologies by implying that scientists are responsible and therefore capable of self-regulating (Hartley et al., 2021). Buzzwords of responsibility, caution, and transparency moved the focus of the debate away from controversial issues, such as uncertainty and unintended consequences to those that all sides of the debate can agree on, like the need to be responsible, cautious, and transparent in scientific work. They also attenuated perceptions of threat posed by gene drive by suggesting that this novel and potentially dangerous technology was ultimately in the safe hands of responsible scientists. However, while they might illuminate the key features of social responsibility of scientists (Douglas, 2003; Loroño-Leturiondo & Davies, 2018), buzzwords may also act like slogans nudging publics to accept the moral authority of scientists and potentially obscure issues of oversight and accountability.

## Conclusion

This analysis reveals a new focus on responsibility in media reporting emanating from the gene drive community. Whether intentional or not, this new focus builds consensus around gene drive by emphasizing the moral authority of the scientific community through the evocation of moral attributes, especially scientific responsibility, caution, transparency, and commitment to democratic values. While in previous debates on emerging technologies, the authority of scientists centered on their knowledge and expertise, a new “moral authority” has become increasingly visible in the media through references to the values shared by scientists and society. This new focus suggests scientists are not seen as separate from a lay society through their expertise but rather are part of society, working *with* lay publics, listening to their views and acting on them (Glerup & Horst, 2014). We have shown that the gene drive community’s evocation of responsibility, caution, and other moral attributes is reflected in the media’s language of gene drive and in promoting the trustworthiness of the gene drive community in the press. It remains to be seen whether this new focus on responsibility will help navigate the controversy surrounding gene drive research.

## Notes

1 Gene drive is a new scientific term with varied and confusing uses (see Alphey et al., 2020), but it has already entered general vocabulary and has been recorded by Lexico (n.d.), an online offshoot of the Oxford English Dictionary. We use the term “gene drive” to refer to this new genetic technology. We also use “gene drive” or “gene drives” to denote a specific genetic modification or modifications.
